# Psychological Well-Being and Quality of Life in Laryngeal Cancer Patients across Tumor

**DOI:** 10.3390/jcm13206138

**Published:** 2024-10-15

**Authors:** Maria Octavia Murariu, Eugen Radu Boia, Delia Ioana Horhat, Cristian Ion Mot, Nicolae Constantin Balica, Cosmin Iosif Trebuian, Alexandru Cristian Brici, Delia Elena Zahoi

**Affiliations:** 1 Department of Doctoral Studies, “Victor Babes” University of Medicine and Pharmacy Timisoara, Eftimie Murgu Square No. 2, 300041 Timisoara, Romania; octavia.brici@umft.ro; 2 ENT Department, “Victor Babes” University of Medicine and Pharmacy Timisoara, Eftimie Murgu Square No. 2, 300041 Timisoara, Romania; eugen.boia@umft.ro (E.R.B.); horhat.ioana@umft.ro (D.I.H.); mot.ion@gmail.com (C.I.M.); 3 Department of Surgery, Emergency Discipline, “Victor Babes” University of Medicine and Pharmacy Timisoara, 300041 Timisoara, Romania; trebuian.cosmin@umft.ro; 4 Emergency Unit, County Emergency Hospital Resita, 320210 Resita, Romania; alex.brici@gmail.com; 5 Department of Anatomy and Embryology, “Victor Babes” University of Medicine and Pharmacy Timisoara, 300041 Timisoara, Romania; zahoi.delia@umft.ro

**Keywords:** laryngeal cancer, psychological impact, quality of life

## Abstract

**Background/Objectives:** Laryngeal cancer significantly impacts patients’ psychological well-being and quality of life (QoL). This study aims to evaluate the psychological impact and QoL in patients with laryngeal cancer, focusing on differences based on tumor stage and treatment. **Methods:** This longitudinal study included 75 patients diagnosed with laryngeal cancer. Participants were assessed at diagnosis and 3 months post-treatment using validated tools such as the Hospital Anxiety and Depression Scale (HADS) and the EORTC QLQ-H&N35 questionnaire. This study analyzed the impact of tumor stage, treatment type, and demographic factors on psychological well-being and QoL. **Results:** Patients with early-stage tumors (Stage I) reported significantly better psychological well-being and QoL compared to those with more advanced tumors (Stages III and IV) both before and after treatment. The non-significant *p*-values in advanced stages suggest a uniformity of severe distress and poor QoL among these patients. Treatment led to significant reductions in anxiety and depression in early-stage patients, while those with advanced-stage disease showed less improvement. **Conclusions:** The findings highlight the critical need for early psychological intervention, particularly in advanced-stage laryngeal cancer patients who continue to experience substantial psychological distress and poor QoL despite treatment. Integrating comprehensive psychological support into standard care is essential to improve overall outcomes for these patients.

## 1. Introduction

Laryngeal cancer is a relatively common malignancy of the head and neck, representing a significant cause of morbidity and mortality worldwide. It accounts for approximately 2–5% of all cancers globally, with an estimated annual incidence of over 180,000 cases and a mortality rate of around 50% due to late-stage diagnoses and aggressive disease progression [1,2]. The majority of laryngeal cancer cases, about 95%, are squamous cell carcinomas, which arise from the epithelial cells lining the larynx [3,4]. Beyond the physical burden, laryngeal cancer has a profound psychological impact on patients. The disease and its treatments often lead to functional impairments, such as difficulties with speaking, swallowing, and breathing, which can severely affect patients’ quality of life. The psychosocial dimensions of living with laryngeal cancer, including anxiety, depression, and social isolation, further exacerbate the burden of the disease. Therefore, understanding the comprehensive impact of laryngeal cancer on patients, both physically and psychologically, is crucial for developing effective therapeutic and supportive care strategies aimed at improving overall patient outcomes [5].

Patients diagnosed with laryngeal cancer frequently experience significant psychological challenges, such as anxiety, depression, and post-traumatic stress disorder (PTSD) [4]. Studies have shown that the prevalence of these psychological conditions is notably high among laryngeal cancer patients, driven by the life-threatening nature of the disease, the potential for significant functional impairments (such as loss of voice and difficulties with swallowing and breathing), and the impact of disfiguring surgical treatments [6,7].

Research has demonstrated that anxiety and depression are particularly common, affecting up to 50% of patients with head and neck cancers, including those with laryngeal cancer. This prevalence is due to concerns about prognosis, recurrence, and the social stigma associated with visible physical changes [8,9]. Additionally, post-traumatic stress symptoms are often observed, as patients may experience traumatic responses related to their diagnosis, invasive treatments, and fear of disease progression. Such psychological distress can profoundly impact overall quality of life, complicate treatment adherence, and hinder the patient’s ability to cope with the illness [10].

Different treatment modalities for laryngeal cancer, such as surgery, radiotherapy, and chemoradiotherapy, have distinct impacts on the patient’s quality of life. Surgical interventions, particularly total laryngectomy, can result in significant functional impairments, including difficulties in communication, swallowing, and breathing [11]. Radiotherapy and chemoradiotherapy may cause side effects like mucositis, xerostomia, and dysphagia, which can further affect daily activities and social interactions [12]. Although these treatments are vital for improving survival rates, they can also lead to substantial psychological challenges, including anxiety and depression due to altered speech and facial disfigurement. This psychological impact is often underestimated in clinical settings, underscoring the need for comprehensive post-treatment support [13].

There is a serious need for systematic psychological evaluation to better understand the psychosocial challenges faced by laryngeal cancer patients. Longitudinal studies, such as the one proposed here, are essential for identifying critical periods where psychological and rehabilitative interventions are most effective. These evaluations can help in developing tailored interventions that address the unique psychological needs of patients, ultimately improving overall outcomes and quality of life.

In this study, we aimed to assess two key areas affected by laryngeal cancer: psychological well-being and quality of life (QoL). To evaluate psychological impact, we employed the Hospital Anxiety and Depression Scale (HADS), which is specifically designed to measure anxiety and depression levels in medical populations. For assessing the impact on QoL, we used the EORTC QLQ-H&N35, a validated questionnaire developed for head and neck cancer patients, addressing critical aspects such as pain, swallowing difficulties, and speech problems. By focusing on these two widely accepted tools, we provide a focused and comprehensive analysis of the psychological and functional challenges faced by laryngeal cancer patients. This approach allows for an in-depth understanding of how cancer and its treatment affect patients’ mental health and overall well-being.

## 2. Materials and Methods

### 2.1. Study Design

This study is a longitudinal, prospective analysis conducted between October 2023 and August 2024 to assess the psychological impact and quality of life in patients diagnosed with laryngeal cancer. This study includes 75 patients from the ENT Clinic Timisoara, who were followed from diagnosis through long-term recovery.

This study enrolled participants who were diagnosed with laryngeal cancer and met specific inclusion and exclusion criteria. Eligible participants included adult patients aged 18 years and older, who were diagnosed with squamous cell carcinoma of the larynx, confirmed through histopathological analysis. Informed consent was obtained from all participants prior to their inclusion in this study. To ensure the reliability and validity of this study, certain patients were excluded. These included those with severe comorbidities that could independently influence their quality of life, as well as those unable to complete the necessary questionnaires due to cognitive impairments or language barriers.

**Figure 1:**
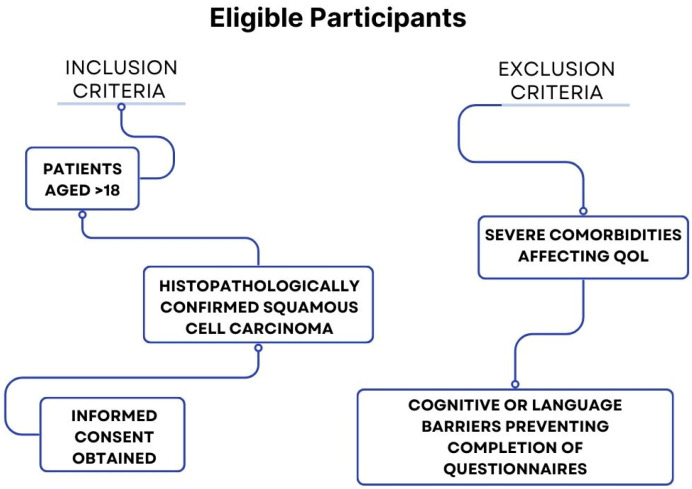
Flowchart showing the inclusion and exclusion criteria for participants in this study on psychological impact and quality of life in laryngeal cancer patients.

For a clearer understanding, the detailed inclusion and exclusion criteria are summarized in Figure 1.

### 2.2. Psychological and Quality-of-Life Assessments

To evaluate the psychological impact and quality of life in the participants, this study employed several validated instruments:

1. Hospital Anxiety and Depression Scale (HADS): HADS is designed to assess the levels of anxiety and depression in patients. It includes 14 items divided into two subscales: anxiety (HADS-A) and depression (HADS-D). Each item is scored from 0 to 3, resulting in a total score range of 0 to 21 for each subscale. Scores of 0–7 indicate normal levels, no anxiety/depression (A/D), 8–10 suggest mild A/D, 11–14 moderate A/D, and 15–21 represent severe A/D.
2. EORTC QLQ-H&N35 (European Organization for Research and Treatment of Cancer Quality of Life Questionnaire-Head and Neck 35): This module is specifically designed for head and neck cancer patients and is used alongside the QLQ-C30. It includes 35 items addressing symptoms such as pain, swallowing difficulties, speech problems, and sensory issues. Similar to the QLQ-C30, scores are standardized on a scale from 0 to 100.

### 2.3. Data Collection Timeline

Data collection was performed at multiple time points to monitor the patients’ psychological and quality-of-life outcomes throughout their treatment and recovery process:

- Pre-Treatment (at diagnosis): initial assessments were conducted to establish baseline psychological and quality-of-life measures.
- Post-Treatment: follow-up assessments were conducted at 3 months post-treatment to track long-term outcomes and recovery patterns.

### 2.4. Statistical Analysis

The data collected in this study were analyzed using MedCalc^®^ Statistical Software version 20.015. Descriptive statistics, including mean, median, standard deviation, and range, were calculated to summarize the demographic and clinical characteristics of the study population. For comparative analysis, the chi-squared test was applied to categorical data. To evaluate changes in HADS-A (anxiety) and HADS-D (depression) scores over time, the Wilcoxon signed-rank test was used, as these variables are ordinal. The paired *t*-test was applied to evaluate changes in EORTC QLQ-H&N35 (quality of life) scores, assuming the data met normality assumptions. A significance threshold of *p* < 0.05 was established, ensuring that the statistical methods employed allowed for the identification of significant trends and correlations between treatment modalities and psychological outcomes.

## 3. Results

This study included 75 patients diagnosed with laryngeal cancer, with demographic and clinical characteristics summarized in Table 1. The average age of the study participants was 63.89 years (SD = 8.88). Age demonstrated a significant influence on quality of life as measured by the EORTC QLQ-H&N35 both before (at diagnostic) and after treatment (at 3 months). However, age was not significantly associated with HADS-A or HADS-D scores, indicating that anxiety and depression levels were relatively consistent across different age groups (*p* > 0.05). When analyzing the data by sex, male patients (61.3% of the cohort) had significantly higher levels of anxiety before treatment compared to females (*p* = 0.033), and this difference persisted post-treatment (*p* = 0.040). Depression and QoL scores did not differ significantly by sex, suggesting that while males experienced higher anxiety, overall QoL and depressive symptoms were similarly affected by sex.

This study also examined the impact of the patients’ living environment (urban vs. rural) on psychological outcomes and QoL. Urban patients (65.3% of the cohort) exhibited marginally higher levels of pre-treatment anxiety compared to rural patients, though this difference was not statistically significant for HADS-A (*p* = 0.053) or HADS-D (*p* = 0.060). Post-treatment, the environment did not show a significant impact on either HADS-A (*p* = 0.061) or HADS-D (*p* = 0.060) scores, nor on QoL as measured by the EORTC QLQ-H&N35 (*p* = 0.025), indicating that the therapeutic interventions likely mitigated any initial differences attributable to environmental factors. Tumor stage at diagnosis was a significant factor influencing both psychological impact and quality of life (QoL). Patients with early-stage tumors (Stage I) reported significantly better QoL and lower levels of anxiety and depression compared to those with more advanced tumors. For Stage I patients, the differences in anxiety (HADS-A), depression (HADS-D), and QoL (EORTC QLQ-H&N35) compared to more advanced stages were all significant (*p* < 0.05). This indicates that patients in Stage I generally experienced less psychological distress and had a higher QoL prior to treatment. As the tumor stage advanced to Stage II, the differences were less pronounced but still notable. In Stage III and Stage IV, the differences were not statistically significant (*p* > 0.05 for all measures), suggesting that patients in these stages experienced uniformly high levels of distress and low QoL. After treatment, the trend persisted but with slightly reduced significance. Stage I patients continued to show better outcomes in anxiety (HADS-A: *p* = 0.010), depression (HADS-D: *p* = 0.015), and QoL (EORTC QLQ-H&N35: *p* = 0.008) compared to more advanced stages. In Stage II, the post-treatment differences remained marginally significant (HADS-A: *p* = 0.055; HADS-D: *p* = 0.050; EORTC QLQ-H&N35: *p* = 0.065). However, for patients in Stage III and Stage IV, the post-treatment *p*-values were non-significant (*p* > 0.05), indicating that the treatment did not substantially change the uniformly poor psychological and QoL outcomes for these patients.

The type of treatment received by patients was another significant determinant of psychological well-being and QoL. Patients who underwent total laryngectomy reported the most substantial declines in QoL, both pre- and post-treatment, with significant differences observed in anxiety and depressive symptoms as well (*p* < 0.05). In contrast, patients who received less invasive treatments, such as laser cordectomy or radiotherapy, exhibited milder psychological impacts and better overall QoL outcomes. Chemoradiotherapy patients showed intermediate results, reflecting the balance between the invasiveness of treatment and its psychological toll.

The results from the comparison of pre-treatment and post-treatment psychological impact and quality-of-life scores are presented below (Table 2). The data reveal significant changes in anxiety, depression, and QoL following treatment for laryngeal cancer.

The mean pre-treatment anxiety score, as measured by the HADS-A scale, was 8.426 (SD = 3.120). Following treatment, the mean anxiety score decreased to 6.870 (SD = 3.050). This decrease in anxiety levels was statistically significant, with a *p*-value of 0.015. The significant decrease in anxiety suggests that the treatment had a positive impact on reducing the psychological burden of anxiety in patients, likely due to the reduction in uncertainty and the start of recovery.

Depression levels, measured by the HADS-D scale, also showed a significant decrease post-treatment. The mean pre-treatment depression score was 10.960 (SD = 4.723), which decreased to 8.450 (SD = 4.570) after treatment. This change was statistically significant, with a *p*-value of 0.022. The decrease in depression scores indicates that patients experienced a moderate improvement in mood and emotional well-being following their treatment, although some level of depression may persist due to the ongoing challenges of living with and recovering from cancer.

Quality of life, as assessed by the EORTC QLQ-H&N35 questionnaire, showed a modest but statistically significant improvement after treatment. The mean pre-treatment QoL score was 61.600 (SD = 25.791), which improved to 55.200 (SD = 24.503) post-treatment. The *p*-value of 0.030 indicates that this improvement was statistically significant. Although patients reported better quality of life post-treatment, the scores suggest that the impact of laryngeal cancer and its treatment on daily functioning and well-being remains substantial, with patients continuing to face challenges in various aspects of their lives.

The analysis stratified by cancer stage and treatment type (Table 3) revealed significant variations in psychological well-being and quality of life (QoL) across different groups. Patients with early-stage tumors (Stage I) who received radiotherapy or partial laryngectomy showed better psychological outcomes and QoL both before and after treatment, with noticeable improvements in HADS-A, HADS-D, and EORTC QLQ-H&N35 scores post-treatment. In contrast, patients with advanced-stage tumors (Stages III and IV), particularly those undergoing total laryngectomy or chemoradiotherapy, exhibited persistently high levels of anxiety and depression, with relatively lower QoL scores, even after treatment. These findings highlight the psychological burden associated with advanced-stage laryngeal cancer, underscoring the need for more comprehensive supportive care in these patients.

The impact of treatment on patients’ psychological well-being and quality of life is well known. These findings underscore the effectiveness of the treatment in improving both psychological well-being and quality of life among laryngeal cancer patients. The significant reductions in anxiety and depression, coupled with the improvement in quality of life, highlight the importance of comprehensive care that addresses both the physical and psychological aspects of cancer treatment.

## 4. Discussion

Laryngeal cancer is a significant health burden, affecting not only patients’ physical well-being but also their psychological health and overall quality of life (QoL). As with many cancers of the head and neck, the treatment of laryngeal cancer often involves aggressive approaches, including surgery, radiotherapy, and chemoradiotherapy, which can have a profound impact on the patient’s ability to speak, swallow, and carry out daily activities. These functional impairments, combined with the emotional burden of a cancer diagnosis, can lead to high levels of anxiety, depression, and a substantial reduction in quality of life [14].

The psychological impact of laryngeal cancer is multifaceted, influenced by factors such as tumor stage at diagnosis, the type of treatment received, and the patient’s demographic characteristics, including age and gender. Early-stage cancer patients may face uncertainty and fear of progression, while those with advanced-stage cancer often struggle with the physical and emotional challenges associated with more invasive treatments and poorer prognoses [15].

Our study revealed significant differences in psychological impact and quality of life (QoL) among patients with laryngeal cancer, particularly when stratified by tumor stage. As expected, patients diagnosed with early-stage tumors (Stage I) demonstrated significantly better psychological well-being and QoL compared to those with more advanced stages (Stage III and IV). This finding aligns with the existing literature, which consistently reports that patients with early-stage cancer generally experience less psychological distress and better overall QoL due to more favorable prognoses and less aggressive treatment requirements [16].

The non-significant *p*-values observed in patients with Stage III and IV tumors, both pre- and post-treatment, suggest a uniformity of severe psychological distress and poor QoL among these patients. This is consistent with the literature that reports a “ceiling effect” in advanced-stage cancer patients, where nearly all individuals experience high levels of distress, making it difficult to detect significant differences within this group [17]. The fact that the treatment did not significantly alter the psychological and QoL outcomes for these patients underscores the need for more targeted psychological interventions and supportive care strategies tailored to those with advanced disease.

Our findings are consistent with previous studies that have documented the profound psychological impact of laryngeal cancer, particularly in advanced stages. For example, Howren et al. (2013) observed similar patterns in head and neck cancer patients, where anxiety and depression levels were significantly higher in patients with advanced-stage tumors [5]. Additionally, the modest improvements in QoL post-treatment observed in our study are in line with research by Anschuetz et al. (2019), who found that while treatment can improve certain aspects of QoL, the challenges associated with advanced laryngeal cancer often persist long after treatment [13].

The significant reduction in anxiety and depression scores after treatment, particularly in early-stage patients, emphasizes the importance of early intervention. These findings suggest that treatment is effective in alleviating psychological distress, at least partially, and improving quality of life in patients with less advanced disease. However, continued poor outcomes in patients with advanced-stage tumors indicate a critical need for ongoing psychological support during treatment and recovery [18].

The results of this study underscore the importance of integrating psychological assessment and support into the standard care of laryngeal cancer patients, particularly for those with advanced-stage disease. Given the high levels of psychological distress and poor QoL observed in these patients, routine screening for anxiety, depression, and QoL should be implemented as part of their care plan. Early identification of psychological distress could lead to timely interventions, potentially mitigating the impact of these symptoms and improving overall patient outcomes [19].

The type of treatment, particularly those that preserve the larynx and voice, plays an important role in determining QoL outcomes in laryngeal cancer patients. Organ-preserving treatments, such as partial laryngectomy or chemoradiotherapy, not only maintain critical functions like speech and swallowing but also have a significant impact on psychological well-being. Studies have shown that preserving the larynx contributes to better long-term QoL and emotional stability, as patients are able to retain their vocal identity and social interactions, which are essential for overall mental health. Therefore, whenever clinically feasible, treatment plans should prioritize organ-preserving approaches to enhance post-treatment QoL and psychological outcomes. This approach aligns with the growing emphasis on not only survival but also the functional and emotional aspects of recovery in head and neck cancer care [20].

While our study provides valuable insights into the psychological impact and QoL outcomes for laryngeal cancer patients, there are several limitations to consider. The sample size, particularly in advanced stages, was relatively small, which may limit the generalizability of our findings. Additionally, while we employed validated tools to assess psychological impact and QoL, these instruments may not capture the full range of psychological experiences in this patient population. One of the limitations of this study is the lack of a control group. The absence of a control group limits our ability to compare the outcomes of the treated patients with those who did not undergo treatment, which could have provided a clearer understanding of the treatment effects. Another limitation of this study is the relatively short follow-up period. Although we measured psychological outcomes and quality of life at two time points (baseline and 3 months post-treatment), a longer follow-up would provide more information about the long-term impact of laryngeal cancer and its treatments. Our future studies should include additional time points, such as 6 months or 1 year, to capture the sustained effects of treatment and disease progression on patients’ mental health and quality of life.

Future research should focus on larger, more diverse patient cohorts to validate these findings and explore the effectiveness of different psychological interventions tailored to laryngeal cancer patients at various stages of disease. Longitudinal studies that extend beyond the 3-month post-treatment period could also provide more comprehensive insights into the long-term psychological and QoL outcomes for these patients.

## 5. Conclusions

This study demonstrates the significant psychological burden and reduction in quality of life (QoL) experienced by patients with laryngeal cancer, particularly as the disease progresses to more advanced stages. Our findings show that patients with early-stage tumors (Stage I) experience relatively better psychological well-being and QoL both pre- and post-treatment, with noticeable improvements following treatment. In contrast, patients with advanced-stage tumors (Stages III and IV) continue to experience substantial psychological distress and poor QoL, despite undergoing treatment. Specifically, the lack of significant improvement in anxiety and depression scores (HADS-A and HADS-D) among these patients post-treatment suggests that current therapeutic approaches may not fully address the psychological complexities of advanced-stage disease. These findings emphasize the critical need for comprehensive care that addresses not only the physical aspects of laryngeal cancer but also prioritizes psychological support. Adapted psychological interventions and supportive care strategies, particularly for those with advanced stage disease, are essential for improving patients’ overall outcomes and quality of life.

## Figures and Tables

**Table 1:** Comparison of anxiety (HADS-A), depression (HADS-D), and quality of life (EORTC QLQ-H&N35) pre- and post-treatment by demographic and clinical characteristics in laryngeal cancer patients.

		Pre-Treatment			Post-Treatment		
Characteristic	Total (N = 75) (%)	HADS-A	HADS-D	EORTC QLQ-H&N35	HADS-A	HADS-D	EORTC QLQ-H&N35
		(Mean ± SD); *p*-Value					
Age		12.1 ± 3.2; 0.112	13.3 ± 3.5; 0.580	56.4 ± 7.1; 0.022	9.0 ± 2.7; 0.130	10.2 ± 3.0; 0.600	48.5 ± 6.3; 0.018
44–50	12 (16%)	8.9 ± 1.7	9.4 ± 2.0	45.6 ± 5.1	6.3 ± 1.8	7.5 ± 2.1	39.8 ± 4.9
51–60	20 (26.7%)	11.2 ± 2.4	12.6 ± 2.9	53.7 ± 6.2	8.8 ± 2.2	10.1 ± 2.6	45.5 ± 5.4
61–70	28 (37.3%)	14.5 ± 2.6	15.8 ± 3.2	62.8 ± 7.3	11.5 ± 2.4	13.2 ± 2.9	56.7 ± 6.9
71+	15 (20%)	17.3 ± 3.0	18.9 ± 3.8	72.1 ± 8.5	15.1 ± 3.0	16.4 ± 3.7	65.3 ± 7.8
Gender							
Male	46 (61.3%)	12.7 ± 2.3; 0.033	14.1 ± 2.8; 0.181	58.3 ± 6.5; 1.000	9.6 ± 2.1; 0.040	10.9 ± 2.6; 0.200	50.2 ± 5.8; 0.900
Female	29 (38.7%)	10.3 ± 2.1; 0.307	11.5 ± 2.5; 0.313	51.2 ± 5.7; 1.000	7.9 ± 2.0; 0.320	9.1 ± 2.3; 0.330	44.1 ± 5.1; 0.950
Environment							
Urban	49 (65.3%)	11.5 ± 2.4; 0.053	12.8 ± 2.7; 0.961	54.1 ± 6.3; 1.000	8.7 ± 2.1; 0.050	10.2 ± 2.5; 0.950	46.7 ± 5.4; 0.980
Rural	26 (34.7%)	13.2 ± 2.5; 0.616	14.5 ± 3.0; 0.449	59.7 ± 7.0; 1.000	10.1 ± 2.2; 0.610	11.4 ± 2.8; 0.470	51.3 ± 6.2; 0.990
Tumor Stage							
Stage I	13 (17.3%)	7.5 ± 1.8; 0.003	8.2 ± 2.1; 0.004	40.5 ± 5.3; 0.005	5.2 ± 1.5; 0.010	6.4 ± 2.0; 0.015	35.2 ± 4.7; 0.008
Stage II	26 (34.7%)	10.4 ± 2.3; 0.050	11.0 ± 2.9; 0.045	50.3 ± 6.7; 0.060	8.3 ± 2.1; 0.055	9.0 ± 2.5; 0.050	42.8 ± 5.4; 0.065
Stage III	29 (38.7%)	14.1 ± 2.5; 0.200	15.5 ± 3.2; 0.150	60.7 ± 7.8; 0.180	12.2 ± 2.4; 0.210	13.8 ± 2.8; 0.160	55.3 ± 7.1; 0.190
Stage IV	7 (9.3%)	18.9 ± 3.1; 0.450	20.3 ± 4.1; 0.300	75.6 ± 9.3; 0.400	16.7 ± 3.0; 0.460	18.5 ± 3.9; 0.320	68.4 ± 8.6; 0.420
Treatment Type							
Total Laryngectomy	20 (26.7%)	12.4 ± 2.3; 0.050	13.7 ± 2.8; 0.180	58.2 ± 6.5; 0.020	9.1 ± 2.0; 0.045	10.5 ± 2.6; 0.190	48.7 ± 5.3; 0.018
Partial Laryngectomy	15 (20.0%)	10.2 ± 2.1; 0.065	11.4 ± 2.5; 0.220	50.4 ± 5.8; 0.025	7.8 ± 1.9; 0.060	8.9 ± 2.3; 0.230	42.3 ± 5.0; 0.022
Laser Cordectomy	10 (13.3%)	8.7 ± 1.9; 0.080	9.6 ± 2.2; 0.240	45.3 ± 5.0; 0.030	6.5 ± 1.8; 0.075	7.5 ± 2.1; 0.250	38.9 ± 4.5; 0.028
Radiotherapy	15 (20.0%)	11.5 ± 2.4; 0.070	12.8 ± 2.7; 0.200	55.1 ± 6.3; 0.035	8.7 ± 2.1; 0.065	10.2 ± 2.5; 0.210	47.4 ± 5.7; 0.032
Chemoradiotherapy	15 (20.0%)	13.9 ± 2.7; 0.055	15.3 ± 3.1; 0.190	62.5 ± 7.2; 0.040	10.5 ± 2.3; 0.050	12.1 ± 2.9; 0.200	54.6 ± 6.8; 0.038

**Table 2:** Changes in anxiety (HADS-A), depression (HADS-D), and quality of life (EORTC QLQ-H&N35) pre- and post-treatment in laryngeal cancer patients.

Measure	Pre-Treatment (Mean ± SD)	Post-Treatment (Mean ± SD)	*p*-Value
HADS-A (Anxiety)	8.426 ± 3.120	6.870 ± 3.050	0.015
HADS-D (Depression)	10.960 ± 4.723	8.450 ± 4.570	0.022
EORTC QLQ-H&N35	61.600 ± 25.791	55.200 ± 24.503	0.030

**Table 3:** Stratified analysis of anxiety (HADS-A), depression (HADS-D), and quality of life (EORTC QLQ-H&N35) scores by cancer stage and treatment type, with the number and percentage of patients in each group. Values are presented as mean ± standard deviation.

Stage	Treatment Type	Total n (%)	HADS-A Pre (Mean ± SD)	HADS-D Pre (Mean ± SD)	EORTC QLQ-H&N35 Pre (Mean ± SD)	HADS-A Post (Mean ± SD)	HADS-D Post (Mean ± SD)	EORTC QLQ-H&N35 Post (Mean ± SD)
Stage I	Radiotherapy	10 (13.3%)	8.5 ± 1.7	9.2 ± 2.0	45.2 ± 5.4	6.1 ± 1.6	7.1 ± 1.9	38.9 ± 4.9
Stage I	Partial Laryngectomy	5 (6.7%)	8.1 ± 1.8	8.9 ± 1.9	44.1 ± 5.2	6.5 ± 1.7	7.3 ± 2.0	39.5 ± 5.0
Stage I	Laser Surgery	3 (4.0%)	7.8 ± 1.5	8.7 ± 1.8	42.9 ± 4.9	6.3 ± 1.5	7.0 ± 1.8	37.8 ± 4.6
Stage II	Radiotherapy	8 (10.7%)	10.2 ± 2.0	11.1 ± 2.5	51.2 ± 5.8	8.0 ± 2.1	9.1 ± 2.3	43.7 ± 5.5
Stage II	Partial Laryngectomy	7 (9.3%)	10.0 ± 2.1	10.7 ± 2.4	50.8 ± 6.0	7.8 ± 2.0	8.9 ± 2.1	42.5 ± 5.2
Stage II	Laser Surgery	5 (6.7%)	9.7 ± 2.0	10.5 ± 2.3	49.5 ± 5.6	7.6 ± 1.9	8.7 ± 2.0	41.9 ± 5.0
Stage III	Total Laryngectomy	12 (16.0%)	13.9 ± 2.6	14.9 ± 3.1	61.4 ± 6.8	11.3 ± 2.4	12.5 ± 2.9	55.0 ± 6.3
Stage III	Chemoradiotherapy	10 (13.3%)	14.1 ± 2.7	15.1 ± 3.3	60.8 ± 6.9	11.5 ± 2.5	12.7 ± 2.9	54.5 ± 6.5
Stage IV	Total Laryngectomy	10 (13.3%)	17.2 ± 3.1	18.8 ± 3.6	71.8 ± 8.0	14.5 ± 2.9	16.3 ± 3.5	66.0 ± 7.5
Stage IV	Chemoradiotherapy	15 (20.0%)	17.5 ± 3.0	19.0 ± 3.8	70.9 ± 7.9	14.7 ± 3.0	16.5 ± 3.7	65.2 ± 7.8

## Data Availability

The original contributions presented in the study are included in the article, further inquiries can be directed to the corresponding authors.
